# The effect of ursodeoxycholic acid in liver functional restoration of patients with obstructive jaundice after endoscopic treatment: a prospective, randomized, and controlled study

**DOI:** 10.1186/1471-2482-13-38

**Published:** 2013-09-22

**Authors:** Enver Fekaj, Arben Gjata, Mehmet Maxhuni

**Affiliations:** 1 Department of Abdominal Surgery, University Clinical Centre of Kosovo, Rrethi i Spitalit, str: p.n., Pristina 10000, Republic of Kosovo; 2 Department of Surgery, University Clinical Centre, “Nene Tereza”, Rruga Dibres 370, Tirana, Albania

**Keywords:** Obstructive jaundice, Ursodeoxycholic acid, Treatment with ursodeoxycholic acid

## Abstract

**Background:**

In patients with obstructive jaundice, multi-organ dysfunction may develop.

**Methods/Design:**

This trial is a prospective, open-label, randomized, and controlled study with the objective to evaluate the effect of ursodeoxycholic acid in liver functional restoration in patients with obstructive jaundice after endoscopic treatment. The aim of this study is to evaluate the effect of ursodeoxycholic acid in liver functional restoration of patients with obstructive jaundice after endoscopic treatment. The hypothesis of this trial is that patients with obstructive jaundice, in which will be administered UDCA, in the early phase after endoscopic intervention will have better and faster functional restoration of the liver than patients in the control group.

Patients with obstructive jaundice, randomly, will be divided into two groups: (A) test group in which will be administered ursodeoxycholic acid twenty-four hours after endoscopic procedure and will last fourteen days, and (B) control group.

Serum-testing will include determination of bilirubin, alanine transaminase, aspartate transaminase, gama-glutamil transpeptidase, alkaline phosphatase, albumin, and cholesterol levels. These parameters will be determined one day prior endoscopic procedure, and on the third, fifth, seventh, tenth, twelfth and fourteenth days after endoscopic intervention.

**Discussion:**

This trial is a prospective, open-label, randomized, and controlled study to asses the effect of ursodeoxycholic acid in liver functional restoration of patients with obstructive jaundice in the early phase after endoscopic treatment.

**Trial registration:**

ClinicalTrials.gov, NCT01688375

## Background

The most common causes of obstructive jaundice are choledocholithiasis, strictures of the biliary tract, cholangiocarcinoma, carcinoma of pancreas, and pancreatitis.

First-line serum testing in a patient with obstructive jaundice should include determination of bilirubin (total and direct fractions), aspartate transaminase (AST), alanine transaminase (ALT), gama-glutamil transpeptidase, and alkaline phosphatase levels [1].

In a prolonged obstruction, multi-organ dysfunction including renal failure, cardiac dysfunction, pulmonary dysfunction, poor hepatic metabolism and hemostasis impairment may develop [2].

It is the conventional concept that the high level of serum bilirubin may cause multisystemic damage in patients with obstructive jaundice. Current pathophysiological studies on obstructive jaundice have shown that the damage to the liver, kidney, and immune system of the patients are closely related to endotoxemia. The key event in the pathophysiology of obstructive jaundice- associated complications is endotoxemia of gut origin because of intestinal barriere failure.

Biliary tract obstruction can cause damage to liver cells and Kupffer cell function. In obstructive jaundice hyper secretion of TNF (tumour necrosis factor) by Kupffer cells may support the production of systemic cytokine and be responsible for significant complications [3-5].

Patients with obstructive jaundice frequently suffer haemodynamic and body fluid disturbances. Acute renal failure is a relevant complication in obstructive jaundice. The extra cellular water volume depletion and myocardial dysfunction affects haemodynamic and renal disturbance in patients with obstructive jaundice [6].

Experimental and clinical studies have suggested that a period of at least four to six weeks is needed for the restoration of normal major synthetic and clearance functions of the liver, as well as mucosal intestinal barrier functions [7].

Even though there is a general assumption about the increased bleeding tendency in obstructive jaundiced patients, it was demonstrated tendency for hyper coagulation independent of increased prothrombin times. The most probable cause of this effect is the increased activity of fibrin polymers on platelet membrane [8].

It is important to emphasize that the most patients with obstructive jaundice require appropriate endoscopic and surgical procedures for their treatment [1].

There are a many studies, where ursodeoxycholic acid (UDCA) was administrated in patients with cholestatic liver diseases.

UDCA was discovered, by Hammerstein in 1902, as the principal bile acid in the polar bear [9].

It is used for the dissolution of cholesterol-rich gallstones in patients with functioning gallbladders, and in the treatment of primary biliary cirrhosis [10,11].

Mechanisms underlying the beneficial therapeutic effects of UDCA on cholestatic liver diseases include: 1) protection of injured cholangiocytes against toxic effects of bile acids, 2) stimulation of impaired biliary secretion, 3) protection of hepatocytes against bile acid-induced apoptosis, 4) immunomodulatory properties with reduce immune-related liver damage [12-15].

In current literature, there are no a randomized, controlled trials testing the effect of UDCA in liver functional restoration of patients with obstructive jaundice after endoscopic treatment.

### Hypothesis

The hypothesis of this trial is that patients with obstructive jaundice, in which will be administered UDCA, in the early phase after endoscopic intervention will have better and faster functional restoration of the liver than patients in the control group.

### Aim and purpose of the research

#### General aim

The aim of this study is to evaluate the effect of UDCA in liver functional restoration of patients with obstructive jaundice in the early period after endoscopic intervention.

### Specific aims

Specific aims are:

– To evaluate the effect of UDCA in relation to the aetiology of obstructive jaundice.

– To assess that in which functional parameters of the liver, treatment with UDCA will have greater impact.

## Methods/Design

### Study objectives

This trial will be a prospective, open-label, randomized, and controlled study. The objective will be to evaluate the effect of ursodeoxycholic acid (UDCA) in the functional restoration of the liver in patients with obstructive jaundice in the early post-endoscopic phase.

### Study design

After diagnosis, patients with obstructive jaundice will be divided into two groups: (A) the test group in which will be administered UDCA in the early phase after endoscopic treatment, and (B) control group, in which no treatment will be applied with UDCA.

Diagnostic methods will be biochemical findings, ultrasound examination, endoscopic retrograde cholangio pancreatography (ERCP), CT-scan and magnetic resonance cholangio pancreatography (MRCP).

Serum-testing in patients with obstructive jaundice will include determination of bilirubin (total and direct fractions), alanine transaminase (ALT), aspartate transaminase (AST), gama-glutamil transpeptidase (GGT), alkaline phosphatase, albumin, and cholesterol levels. These parameters will be determined one day prior endoscopic intervention, and on the third, fifth, seventh, tenth, twelfth and fourteenth days after endoscopic intervention.

Endoscope procedure that will end with the internal derivation of bile shall be named as the internal bile drainage.

### Inclusion criteria are

– Patients with obstructive jaundice

– Serum bilirubin level higher than 50 μmol/l

– 19+ years of age

– Written informed consent

### Exclusion criteria are

Cholangitis

– Acute pancreatitis

– Pregnant women

– Women during the breastfeeding

– Suspected or proven primary liver diseases

– My family members

– Patients who are unable to understand our study purpose

During our trial, from the study will be excluded patients who show signs of serious side effects and allergic reactions after treatment with UDCA.

### UDCA administration

UDCA administration will begin twenty-four hours after endoscopic procedure and will last fourteen days. UDCA dose will be administered at 750 mg/day, divided into three doses.

### Power of the study

A clinically relevant improvement of liver functional tests is defined as an improvement of 70% of liver functional tests in test group, and an improvement only 40% in control group. In our study, to have an 80% chance of detecting about a 40% difference (70% vs. 40%) between two groups on improvement of liver functional tests at an alpha level of 0.05, the power calculation indicates that each of the two groups should have at least 48 patients.

### Data sources and search strategy

An electronic search was performed on PubMed (from 1 January 1985 to 1 February 2012). A combination of keywords and MeSH terms where: ‘ursodeoxycholic acid’ AND ‘obstructive jaundice’, ‘obstructive jaundice’ AND ‘liver function tests’, ‘obstructive jaundice’ AND ‘acute renal failure’. I have used, also, limits: Type of article (selection was- clinical trial, meta-analyses), Species (selection was- human, animals), Text options (selection was- links to free full text, abstract), Languages (English), Sex (male, female), Age (all adult 19+ years), Field (all fields).

### Outcomes

The primary outcome measure in this trial is liver functional restoration. The secondary outcome is assessment liver functional parameters in which, treatment with UDCA, will have greater impact. Follow-up measures will be collected one day prior endoscopic intervention, and on the third, fifth, seventh, tenth, twelfth and fourteenth days after endoscopic intervention.

### Randomisation

Patients have to sign an informed consent for the involving in the trial a day before endoscopic procedure. Randomisation will be performed at the time of transfer to the endoscope room by using random number generator at http://www.stattrek.com.

### Ethics

This study will be conducted in accordance with the principles of the Declaration of Helsinki. The study protocol has been approved by Ethics and Professional Committee at University Clinical Centre of Kosovo. Informed consent will be obtained from all participants.

### Data collection and statistical analyses

Data including serum-test results will be collected in a computer secured study platform. These data will be collected continuously, for each patient, starting one day before endoscopic procedure until the last data fourteen days after intervention.

X^2^-analysis or Fisher exact test will perform to test the differences in proportions of qualitative variables between groups. Mann Whitney U test and Kruskal Wallis test will use for testing the difference between quantitative variables when distribution is not normal and Student t-test or ANOVA test when distribution is normal. The level P < 0.05 will consider as the cut-off value for significance.

## Discussion

In many studies, it was shown the positive effect of UDCA administration in patient with cholestatic liver desease.

UDCA exerts anti-apoptic effects in experimental in vivo models as well as in vitro in primary human hepatocytes. This anti-apoptic effect may contribute to the alleviation of liver injury during UDCA treatment [16].

After UDCA administration in patients with non-alcoholic fatty liver disease, the results show that UDCA is able to reduce serum levels of hepatic enzymes, but this effect is not related to modifications in liver fat content [17].

The study by Willot S, demonstrates the beneficial effect of UDCA on liver function in children after successful surgery for biliary atresia [18].

UDCA in a dose of 13–15 mg\kg\day should be considered in all patients with primary biliary cirrhosis who have abnormal liver enzymes [19].

Long-term high-dose UDCA therapy is associated with improvement in serum liver tests in primary sclerosing cholangitis, but does not improve survival and was associated with higher rates of serious adverse events [20].

UDCA administration can be considered in cases of nodular regenerative hyperplasia of the liver associated with abnormalities of liver enzymes [21].

It seems that combined therapy with UDCA and polyunsaturated phosphatidylcholine could be considered in obstetric cholestasis [22].

It was demonstrated that ciprofloxacin and UDCA have a synergic effect on prevention of bacterial translocation in obstructive jaundiced patients [23].

This trial is a prospective, open-label, randomized, and controlled study with the objective to evaluate the effect of ursodeoxycholic acid in liver functional restoration in patients with obstructive jaundice after endoscopic treatment. The hypothesis of this trial is that patients with obstructive jaundice, in which will be administered UDCA, in the early phase after endoscopic intervention will have better and faster functional restoration of the liver than patients in the control group.

## Abbreviations

TNF: Tumour necrosis factor; UDCA: Ursodeoxycholic acid; ERCP: Endoscopic retrograde cholangio pancreatography; CT: Computerized tomography; MRCP: Magnetic resonance cholangio pancreatography; ALT: Alanine transaminase; AST: Aspartate transaminase; GGT: Gama-glutamil transpeptidase; HCV: Hepatitis C virus.

## Competing interests

The authors declare that they have no competing interests. This study has not received external funding.

## Authors’ contributions

EF wrote the manuscript and designed the study. AGJ revised the manuscript and co-authored the writing of the manuscript. MM revised the manuscript. All authors have read and approved the final manuscript.

## Pre-publication history

The pre-publication history for this paper can be accessed here:

http://www.biomedcentral.com/1471-2482/13/38/prepub
